# Ultra-deformable liposomes containing terpenes (terpesomes) loaded fenticonazole nitrate for treatment of vaginal candidiasis: Box-Behnken design optimization, comparative *ex vivo* and *in vivo* studies

**DOI:** 10.1080/10717544.2020.1837295

**Published:** 2020-10-28

**Authors:** Rofida Albash, Yasmina Elmahboub, Kholoud Baraka, Menna M. Abdellatif, Ahmed Adel Alaa-Eldin

**Affiliations:** a Department of Pharmaceutics, College of Pharmaceutical Sciences and Drug Manufacturing, Misr University for Science and Technology, Giza, Egypt; b College of Pharmaceutical Sciences and Drug Manufacturing, Misr University for Science and Technology, Giza, Egypt; c Microbiology and Immunology Department, Faculty of Pharmacy, Damanhour University, El Behira, Egypt; d Department of Industrial Pharmacy, College of Pharmaceutical Sciences and Drug Manufacturing, Misr University for Science and Technology, Giza, Egypt; e Department of Pharmaceutics, Faculty of Pharmacy, Fayoum university, Elfayoum, Egypt

**Keywords:** Box Behnken design, fenticonazole nitrate, microbiological study, ultra-deformable liposomes, terpesomes, vaginal drug delivery

## Abstract

Fenticonazole nitrate (FTN) is a potent antifungal drug adopted in the treatment of vaginal candidiasis. It has inadequate aqueous solubility hence, novel ultra-deformable liposomes ‘Terpesomes’ (TPs) were developed that might prevail over FTN poor solubility besides TPs might abstain the obstacles of mucus invasion. TPs were assembled by thin-film hydration then optimized by Box Behnken design utilizing terpenes ratio (X_1_), sodium deoxycholate amount (X_2_), and ethanol concentration (X_3_) as independent variable, whereas their impact was inspected for entrapment efficiency (Y_1_), particle size (Y_2_), and polydispersity index (Y_3_). Design Expert^®^ was bestowed to select the optimal TP for more studies. The optimal TP had entrapment efficiency of 62.18 ± 1.39%, particle size of 310.00 ± 8.16 nm, polydispersity index of 0.20 ± 0.10, and zeta potential of −10.19 ± 0.2.00 mV. Elasticity results were greater in the optimal TP related to classical bilosomes. Further, *ex vivo* permeation illustrated tremendous permeability from the optimal TP correlated to classical bilosomes, and FTN suspension. Besides, *in vivo* assessment displayed significant inhibition effect in rats from FTN-TPs gel compared to FTN gel. The antifungal potency with undermost histopathological variation was detected in rats treated with FTN-TPs gel. Overall, the acquired findings verified the potency of utilizing FTN-TPs gel for treatment of vaginal candidiasis.

## Introduction

Vaginal infections upset women through generating the most prevailing purpose for gynecological examinations. Despite the fact vaginal infections do not endure a great mortality rate, these disorders might cause premature birth, abortion, pelvic inflammations, and transmission of sexual disorders (Martínez-Pérez et al. 2018). *Candida albicans* (*C. albicans*) is usually the causative agent for vaginal infections. For handling of such infection, local application of antifungal bio-actives is eligible to circumvent the drawbacks caused by the systemic administration of antifungal bio-actives (Abdellatif et al. 2020). To accomplish a proficient local conveyance to mucosal tissue, the infiltration into/through the mucus mesh, uniform distribution of drug into the underlying tissue, and sufficiently high drug concentration are required. However, classical vaginal dosage forms as lotion, gels, creams, tablets, soft capsules, pessaries, and solutions have several shortcomings as leakage, messiness and not convenient for the conveyance of slightly soluble drugs to vaginal tissues (Jøraholmen et al. 2017).

Fenticonazole nitrate (FTN) is an antifungal imidazole ring derivative that operates via hindering ergosterol integration, and sequentially destructing the cytoplasmatic outer membrane. FTN is suggested for treatment of mycoses, and vaginal candidiasis (Campos et al. 2018). FTN has fungicidal, and fungistatic actions on fungi, yeasts and dermatophytes. Moreover, it is effective against Gram-positive bacteria (Jung et al. 1988). As many azoles are insoluble as FTN aqueous solubility is <0.10 mg/mL, and this might have a negative influence on its antifungal potency, side effects, pharmacokinetic variability, and the progression of drug resistance. Thus, the techniques to deliver antifungals are crucial for the compelling cure of fungal infections (Yang et al. 2008).

Recently, liposomes have been greatly inspected for enhancing the targeting and therapeutic impacts of slightly soluble bio-actives to vaginal tissues (Antimisiaris & Mourtas 2015). Few studies have mentioned the merits of ultra-deformable liposomes in vaginal drug conveyance. Ultra-deformable liposomes are composed of phospholipids, cholesterol (or no), an edge activator and water. The edge activators could augment the resilience of the phospholipid bilayers. The extremely high flexibility of their membrane allows ultra-deformable liposomes to compress themselves via pores much smaller than their diameter, transporting the entrapped drug into deep tissues (Singh et al. 2017). These merits suggest that ultra-deformable liposomes might be regarded as an appropriate delivery system for their administration on mucosal tissue such as vagina.

Terpenes have acquired great consideration in the pharmaceutical preparations as penetration enhancers (Younes et al. 2018). Also, they might perform as antifungal, and antimicrobial agents due to their accumulation in the lipophilic hydrocarbon molecules of the cell lipid bilayer; such effect permits the easier delivery of essential oils constituents to the inner of the cell (Nazzaro et al. 2017), this produces cytoplasmic infiltration, and cell damage (Jing et al. 2014).

In a view of this, we represent the preparation, and characterization of novel ultra-deformable liposomes ‘terpenes containing ultra-deformable liposomes’ Terpesomes (TPs) for vaginal delivery that might enhance permeation of the antifungal drug through the vaginal mucous membrane, instead of being trapped and more rapidly washed to the outer, luminal layers of the mucus.

To optimize any pharmaceutical formulation, designing a formulation with the optimal features with the least number of experimental runs is the most important consideration. Response-surface-methodology (RSM) might be utilized in the fabrication of new drug delivery systems (Abdelbary & AbouGhaly 2015). Box-Behnken designs (BBD) as types of RSM designs are a category of rotatable or roughly rotatable second-order designs depend on incomplete factorial designs. One more merit of the BBD is that it does not involve combinations for which all factors are concurrently at their extremes. Hence, these designs are convenient in circumventing trials operated at the utmost conditions for which unfavorable outcome might appear (Ferreira et al. 2007).

As far as we know, there is no released data concerning the use of nanocarriers to boost the vaginal delivery of FTN. Thus, the target of the current investigation was to assess the potency of TPs to augment the vaginal permeation of FTN and explore its tolerability. To achieve this target, diverse variables affecting vesicles’ properties were evaluated employing BBD to determine the optimal TP. Terpenes ratio (X_1_), sodium deoxycholate (SDC) amount (X_2_), and ethanol concentration (X_3_) were studied as independent variables, while entrapment efficiency percentage (EE%; Y_1_), particle size (PS; Y_2_), and polydispersity index (PDI; Y_3_) were opted as dependent variables. The optimal TP was assessed for morphology and compared to classical bilosomes (bile salt containing vesicles) in terms of elasticity and *ex vivo* permeation. *In vivo* studies were carried out to compare antifungal potency of FTN-TPs gel compared with FTN gel. Further, histopathological studies were fulfilled to assess the safety of FTN-TPs gel.

## Material and methods

### Materials

Fenticonazole nitrate (FTN) was supplied from Andalous Pharmaceutical Co. (Cairo, Egypt). l-α phosphotidylcholine from egg yolk, sodium deoxycholate (SDC) and ß-estradiol-17-valerate were attained from Sigma Aldrich Chemical Co. (St. Louis, MO, USA). Limonene and citral were purchased from Alfa Aesar (GmbH, Germany). Acetonitrile and methanol (HPLC grade) were obtained from Merck (Darmstadt, Germany). Hydroxypropyl methyl cellulose (HPMC) K4M was obtained from Colorcon (Kent, UK).

### Fabrication of FTN-TPs

TPs were assembled by using limonene and citral at different ratios 1:1, 1:2 and 1:3 (w/w) and SDC (10, 20 and 30 mg) employing thin-film hydration approach (Table 1) (Albash et al. 2019). At first, phospholipid (100 mg), SDC, terpenes and FTN (10 mg) were dissolved in 10 mL methanol in a round flask. By maintaining pressure under vacuum for 30 min, the organic phase was removed at 60 °C utilizing a rotatory evaporator (Rotavapor, Heidolph VV 2000, Burladingen, Germany) at 90 rpm to acquire thin film of TPs. The film was hydrated using 10 mL distilled water enclosing ethanol at different concentrations 5, 10 and 15% (v/v) at 60 °C (Varona et al. 2011). For entire film hydration, glass-beads were utilized for 45 min. After that, TPs were subjected to sonication using bath sonicator (Ultra Sonicator, Model LC 60/H Elma, Germany) for 10 min for additional PS downsizing. Afterwards, the vesicles’ dispersion was kept at 4 °C. For the sake of comparison, FTN-loaded bilosomes (100 mg phospholipid and 10 mg SDC) were prepared as stated except in the hydration stage the film was hydrated with 10 mL distilled water.

**Table 1.** Box-Behnken design for optimization of FTN-loaded TPs.

	Levels		
Factors (independent variables)	Low	Medium	High
	(−1)	0	(+1)
X_1_: Terpenes ratio	1:1	1:2	1:3
X_2_: SDC amount (mg)	10	20	30
X_3_: Ethanol concentration (v/v)	5	10	15
Responses (dependent variables)	Constraints		
Y_1_: EE (%)	Maximize		
Y_2_: PS (nm)	Minimize		
Y_3_: PDI	Minimize		

EE%: entrapment efficiency percent; FTN: fenticonazole nitrate; PS: particle size; PDI: polydispersity index; SDC: sodium deoxy cholate; TPs: terpesomes.

### Characterization of FTN-TPs

#### Calculation of entrapment efficiency percentage (EE%)

The TPs dispersion was centrifugated by a cooling centrifuge (Sigma 3 K 30, Germany) at 20,000 rpm for 1 h at 4 °C. Afterward, the sediment was destroyed employing methanol and assessed at λ_max_ 252 nm by UV-Vis spectrophotometer (Shimadzu UV1650 Spectrophotometer, Koyoto, Japan). EE% was evaluated by applying the subsequent equation (Abdellatif et al. 2017):

(1) $$\text{EE\% }=(\frac{\text{Entraped FTN}}{\text{Total FTN concentration}})\times 100$$

All measurements were performed in triplicate ± SD.

#### Evaluation of particle size (PS), polydispersity index (PDI) and zeta potential (ZP)

PS, PDI, and ZP were assessed for the fabricated TPs by means of a Malvern Zetasizer 2000 (Malvern Instruments Ltd., UK). The measurements were done after suitable dilution (10-fold with de-ionized water) (Abdellatif et al. 2017). The ZP assessment was performed by observing the electrophoretic migration of the vesicles in the electrical field. All measurements were carried out in triplicates.

#### Experimental design construction

BBD was established to check out the influence of diverse factors in fabricating TPs operating Design expert^^®^^ version 11 (Stat Ease, Inc., Minneapolis, MN, USA). The design demanded to construct 15 trials. Three factors were investigated: terpenes ratio (X_1_), SDC amount (X_2_) and ethanol concentration (X_3_) that were elected as independent variables, whereas EE% (Y_1_), PS (Y_2_) and PDI (Y_3_) were elected as dependent variables (Table 1).

#### Selection of the optimal TP

The optimal TP election was counted on the desirability function which granted the inspection of all responses at a similar time. The optimization stage was established to achieve a solution with the least PS and PDI and the highest EE%. The suggestion with the highest desirability solution (near to one) was selected.

#### Transmission electron microscopy (TEM)

Morphology of the optimal TP was observed via TEM (Joel JEM 1230, Tokyo, Japan). One drop without dilution from the optimal TP was arranged as a thin film on a carbon laminated copper grid, stained employing phosphotungstic acid 1.5% (Abdellatif et al. 2017).

#### Differential scanning calorimetry (DSC)

The thermal assessment of FTN and the optimal TP was achieved via differential scanning calorimetry (DSC-60, Shimadzu Corp., Kyoto, Japan) standardized with indium. About 5 mg from samples was placed in aluminum pan and subjected to heat in a temperature range of 10–250 °C at a rate of 5 °C/min under nitrogen stream (25 mL/min) (Albash et al. 2019).

#### Elasticity evaluation

Extrusion technique was utilized to compare elasticity for the classical bilosomes and optimal TP. Vesicles were passed through a filter with a diameter of 200 nm at 2.5 bar (Haug Kompressoren AG; Büchi Labortechnik AG, Flawil, Switzerland). The elasticity of the vesicles was expressed as a percentage change (%) in the size of the vesicles after extrusion (Gaafar et al. 2014). Statistical significance was done by Student’s *t*-test using SPSS^^®^^ software 22.0. The difference at *p* ≤ .05 was recognized significant. The experiment was operated in three times.

### *Ex vivo* studies

### Tissue preparation

The freshly harvested vaginal tube with thickness of 3 ± 6 mm was obtained by opening the abdominal cavity and disarticulated the pubic symphysis (Shabsigh et al. 1999). At first, the vaginal opening was separated from the surrounding perineal skin. Then, the vaginal tube was removed intact. Afterwards, the vaginal tube was sliced to longitudinal parts, and the anterior vaginal wall was obtained. Then the anterior vaginal wall was sliced transversely into proximal and distal parts. The proximal part was soaked in distilled water. *Ex vivo* permeation studies were assessed within 4 h of harvesting the vaginal tissue (Mao et al. 2019).

### *Ex vivo* permeation

*Ex vivo* permeation assessment was carried out using locally fabricated Franz’s diffusion cell with a diffusion area of 0.706 cm^2^. Fresh rats’ vaginal mucosa was fixed between the donor and receptor compartments. Samples (2 mg FTN) from FTN suspension, classical bilosomes, and the optimal TP were placed in the donor cell. The receptor compartment filled with 12 mL of phosphate buffer saline solution (pH 4.5) containing 25% ethanol to establish sink state and maintained at 37 ± 1 °C (Sanz et al. 2018). The system was properly sealed with parafilm. At an appropriate time, 0.5 mL was withdrawn, and replaced by an equal volume of fresh media. The samples were filtered through a 0.45 µm membrane and analyzed by using a validated HPLC method (Quaglia et al. 2001). The permeation flux (J_max_) at 12 h and the enhancement ratio (ER) were assessed by the consecutive equation (Albash et al. 2019):

(2) $$\mathrm{Jmax}\;=(\frac{\text{quantity of FTN infilterted}}{\mathrm{time}\;\times \text{ area of membrane}})$$

(3) $$\mathrm{ER}\;=(\frac{\text{Jmax of the vesicles}}{\text{Jmax of FTN}})$$

The experiment was operated in triplicate ± SD. Statistical significance was determined through one-way ANOVA by SPSS^^®^^ software 22.0. Post-hoc test was performed employing Tukey’s honest significant difference (HSD) test. At the end of the study, the vaginal tissues were removed from the diffusion cell and washed in distilled water for 10 s to remove the adhering drug. The tissues were then cut into small pieces and sonicated in 5 mL methanol for 30 min using bath sonicator to leach out the deposited drug. The samples were analyzed HPLC method.

### Formulation of FTN gel and FTN-TP gel

FTN suspension and the optimal TP were converted into gel to enhance the vaginal retention where a specified amount of HPMC was mixed by the assistance of magnetic stirrer to the optimal TP and FTN suspension to have a final FTN gel concentration of 2% w/w (Abdellatif et al. 2017).

### *In vivo* studies

#### Antifungal study

##### Animals

The study design was certified through approval from ethical board of the Faculty of Pharmacy, Damanhour University (Ref.No.720PM16). Female Sprague Dawley rats weighed 200–250 gm were utilized throughout the entire studies. They were given access to chow and water. They were habituated three per cage and maintained at 21 °C and 50–55% humidity under a 12:12 h light-dark cycle. Prior infection, all rats were subcutaneously injected with estradiol valerate dissolved in 0.10 µL sesame oil and given in a dose of 0.5 mg for each rat for six days to induce pseudo estrous (De Bernardis et al. 1997). Moreover, rats were administrated with Dexamethasone (Fortecortin; Merck Laboratories) (1 mg/L) as immunosuppressant and Amoxycillin (SAIMED innovation) (250 mg/L) in drinking water and continued through experiment (Martinez et al. 2001).

##### Vaginal candidiasis

One week after 1^st^ estradiol valerate injection, all subjects were injected intravaginally with *C.albicans* cultures dissolved in 0.10 µL of saline by automatic micropipette, the control rats were inoculated intravaginally with saline, all the rats were left undisturbed for 4 days. On day 5 post-infection, rats were washed vaginally with 500 µL of saline and stained with Gram stain to assert the infection in all rats by existence of characteristic shape of *C. albicans* under microscope. The fungal suspension was diluted and cultured on Sabouraud dextrose agar. The colony forming units (CFU) was estimated and the total CFU per vaginal sample was calculated. Animals were weighed before and after infection as well as after treatment. No weight loss was discovered in any of the groups. This illustrated that particle administration did not lead to overt weight loss and subsequently toxicity. This observation was anticipated after candidiasis infection since the infection is asymptomatic in mice (Lucena et al. 2018).

##### Antifungal therapy

The treatment began 4 days following the infection and continued for 7 days by administrating 20 µL from each treatment by micropipette. Twenty-four rats were classified into four groups where group I acted as normal control, group II acted as positive control and kept untreated, group III treated with FTN gel and group IV treated with optimal FTN-TPs gel. The results were reported as the average of three sets of values. Statistical significance was studied using one-way ANOVA adopting SPSS^^®^^ software 22.0. Post-hoc test was conducted using Tukey’s HSD test.

### *In vivo* histopathological assessment

After scarification, the vaginal tissues were isolated, fixed and sliced by a microtome (Leica Microsystems SM2400, Cambridge, UK) (Kim et al. 2010). Each slide was examined by the following parameters: existence of fungal cells, deterioration of the epithelium, infiltration of inflammatory cells, vacuolization and edema.

## Results and discussion

### Optimization of BBD

RSM relies on experimental design with the aim of estimating the optimal variables for a definite goal of the response, using minimum experiments (Aboelazayem et al. 2018). The fabricated TPs were optimized through applying BBD employing Design-expert^^®^^ software that developed 15 experimental runs with three center points. The model opted was linear model for EE% and was quadratic model for both PS and PDI. Adequate precision is utilized to assert that the model could be opted to navigate the design space. A ratio greater than four is favored which was observed for all dependent variables as depicted in Table 2. The predicted R^2^ values were in a desired agreement with the adjusted R^2^ in all dependent variables (Table 2). The negative value of the predicted R^2^ value of PDI justifies that the mean is a better presenter of the response (Albash et al. 2019).

**Table 2.** Results of regression analysis for responses (Y_1_) EE%, (Y_2_) PS and (Y_3_) PDI.

	Model	Adequate precision	R^2^	Adjusted R^2^	Predicted R^2^	*p* Value
Y_1_: EE%	Linear	18.56	0.90	0.87	0.80	<.0001
Y_2_: PS (nm)	Quadratic	24.93	0.99	0.97	0.86	.0001
Y_3_: PDI	Quadratic	5.92	0.79	0.43	−2.91	.19

EE%: entrapment efficiency percent; PS: particle size; PDI: polydispersity index.

### Impact of formulation variables on the EE%

**Figure 1.**
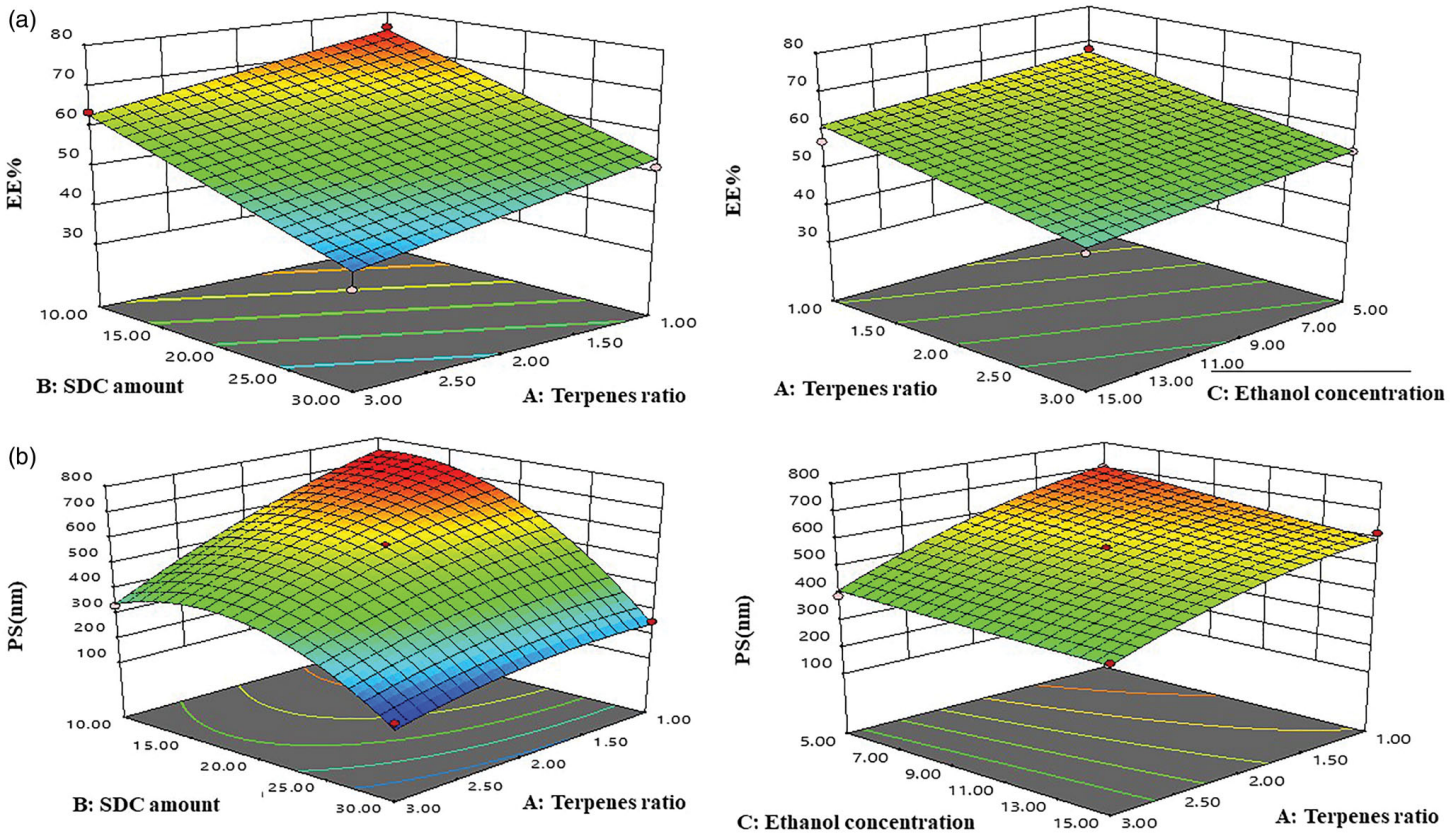
Response 3D plots for the effect of terpenes ratio (X_1_), SDC amount (X_2_) and ethanol concentration (X_3_) on (a) EE% and (b) PS. SDC: sodium deoxy cholate; EE%: entrapment efficiency percent and PS: particle size.

**Figure 2.**
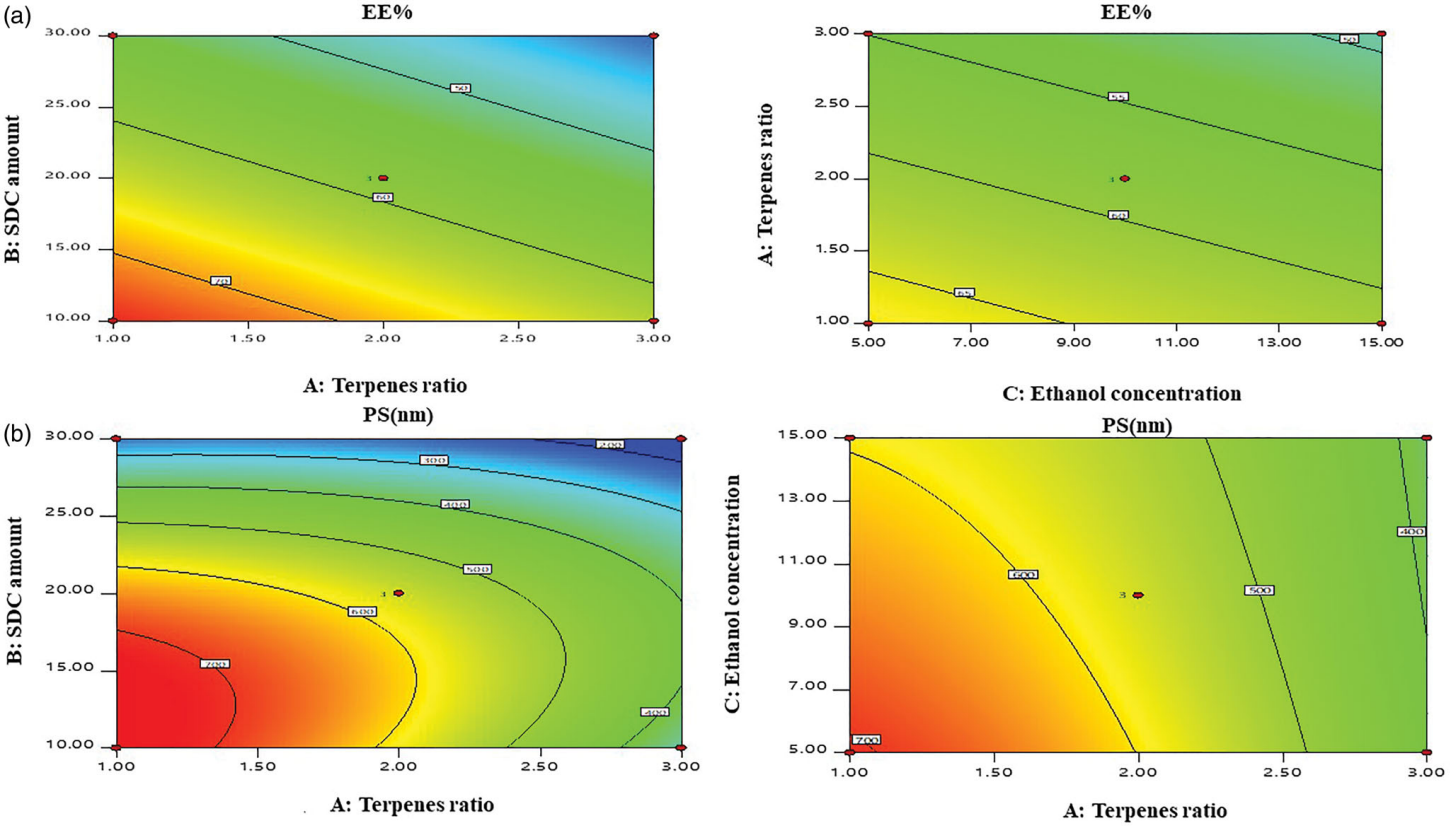
Contour plots for the effect of terpenes ratio (X_1_), SDC amount (X_2_) and ethanol concentration (X_3_) on (a) EE% and (b) PS. SDC: sodium deoxy cholate; EE%: entrapment efficiency percent and PS: particle size.

As depicted in Figures 1(a) and 2(a), EE% ranged from 37.08 ± 0.69% to 75.83 ± 2.04% (Table 3). Linear model was the perfect model for the investigation of the gained data. The results declared that augmenting terpenes ratio (X_1_) had a significant (*p* = .0005) negative influence on EE% our findings were in a agreement with Prasanthi & Lakshmi (2013). In addition, Dragicevic-Curic et al. (2011) stated that the inclusion of terpenes intensifies the fluidity of the phospholipid encircling the C_16_ atom of phospholipid acyl chains that might deteriorate TPs integrity to enclose FTN. Also the type of terpene and its proportions in terpenes mixture significantly affected FTN EE% where reducing the proportions of lipophilic terpene limonene (log *P* = 4.83) from 1:1 to 1:2 and 1:3 in terpenes mixture and with subsequent increase in proportion of less lipophilic terpene citral (log *P* = 2.02) led to reduction in FTN EE%. The higher the lipophilicity of the terpene, the higher solubilization of the lipophilic drug (FTN), and hence the higher EE% owing to an increased space for the drug incorporation in the lipid bilayer resulting in high EE% (Mura et al. 2009). On the other hand, additional increase in SDC amount (X_2_) decreased EE% significantly (*p* < .0001) and resulted in pore formation in the bilayers of TPs (Aboud et al. 2016). Moreover, increasing ethanol concentration (X_3_) in the TPs resulted in lowering EE% significantly (*p* = .0423), this might be associated with increased fluidity and infiltration of the vesicular membrane leading to FTN depletion from the TPs during fabrication (Faisal et al. 2018).

**Table 3.** Composition of three level three factor Box Behnken design of FTN-loaded TPs and EE%, PS, PDI and ZP results.

Run	X_1_ (Terpenes ratio)	X_2_ (SDC amount)	X_3_ (Ethanol concentration)	EE%	PS (nm)	PDI	ZP (mV)
Mid points							
TP1	1:1	30	10	51.36 ± 0.92	251.10 ± 1.39	0.58 ± 0.06	−7.88 ± 0.16
TP2	1:3	30	10	37.08 ± 0.69	168.13 ± 6.02	0.56 ± 0.009	−6.82 ± 0.14
TP3	1:1	10	10	75.83 ± 2.04	724.15 ± 9.95	0.56 ± 0.04	−6.70 ± 0.20
TP4	1:3	10	10	63.52 ± 1.33	331.80 ± 9.31	0.56 ± 0.02	−9.93 ± 0.32
TP5	1:1	20	5	67.90 ± 0.55	700.96 ± 14.82	0.72 ± 0.04	−5.03 ± 0.26
TP6	1:3	20	5	54.71 ± 0.27	391.73 ± 10.30	0.53 ± 0.008	−6.70 ± 0.20
TP7	1:1	20	15	57.08 ± 0.69	617.43 ± 41.00	0.62 ± 0.02	−9.70 ± 0.21
TP8	1:3	20	15	47.77 ± 1.34	388.73 ± 3.30	0.49 ± 0.008	−8.81 ± 0.65
TP9	1:2	10	5	71.29 ± 0.76	683.2 ± 16.8	0.44 ± 0.50	−12.43 ± 0.30
TP10	1:2	30	5	55.20 ± 1.75	235.55 ± 1.35	0.45 ± 0.01	−10.83 ± 0.28
TP11	1:2	10	15	70.23 ± 0.76	521.36 ± 8.19	0.35 ± 0.03	−10.76 ± 0.09
TP12	1:2	30	15	51.13 ± 0.48	197.56 ± 0.49	0.56 ± 0.01	−9.00 ± 0.18
Center points							
TP13	1:2	20	10	56.70 ± 0.35	566.03 ± 17.64	0.50 ± 0.01	−5.90 ± 0.08
TP14	1:2	20	10	56.63 ± 0.45	556.33 ± 18.62	0.52 ± 0.008	−5.50 ± 0.29
TP15	1:2	20	10	56.78 ± 0.70	557.66 ± 11.14	0.51 ± 0.009	−5.86 ± 0.12

EE%: entrapment efficiency percent; FTN: fenticonazole nitrate; PS: particle size; PDI: polydispersity index; SDC: sodium deoxy cholate; TPs: terpesomes; ZP: zeta potential.

### Impact of formulation variables on PS

According to literature, the optimum PS range to intensify retention of vesicles inside vaginal tissue ranges from 200 to 800 nm. Displaying size inside this range, vesicles might pass through cervicovaginal mucus without revealing the drug to the systemic circulation (De Abreu et al. 2016). As illustrated in Figures 1(b) and 2(b), the PS ranged from 168.13 ± 6.02 to 724.15 ± 9.95 nm showing that TPs intended for topical application in the vagina is with a low liability to cause systemic toxicity (Table 3). A quadratic model was perfect for the analysis of the gained results. Regarding terpenes ratio (X_1_) it was found by changing the ratio of limonene:citral from 1:1 to 1:3 reduced the PS of TPs significantly (*p* < .0001). The PS measurements displayed comparable findings to EE% results. TPs fabricated using 1:1 as terpenes ratio had the greatest PS followed by 1:2 then 1:3, this could be associated with there is a direct relevance that correlates PS of the TPs with the amount of drug enclosed. Besides, high drug entrapment expands the space among the vesicular bilayers because of drug insertion in the hydrophobic zones inside the vesicles so TPs with the highest EE% will obtain the greatest PS (El-Nabarawi et al. 2020). ANOVA findings showed SDC amount (X_2_) had a significant influence on PS (*p* < .0001) at greater SDC amount, the surface tension was reduced, aiding in the formation of smaller TPs. Albash et al. (2019) also published similar findings in their investigation on the preparation of olmesartan medoxomil transethosomes they reported that augmenting the amount of SDC participated in the formation of significantly small vesicles owing to micelles formation. Furthermore, the PS of the prepared TPs decreased significantly (*p* = .0191) by increasing the concentration of ethanol (X_3_) from 5% to 15% our results were in accordance with Dragicevic-Curic et al. (2009). At a higher concentration of ethanol, the thickness of the membrane is markedly decreased, that leads to the production of interpenetrating hydrocarbon chain phase (Imam et al. 2017). Another fact behind the decreased PS is due to modification of the surface charge of ethanol that confers some degree of stabilization and thus, it may finally decrease the PS (Madhavi et al. 2013).

### Impact of formulation variables on PDI

In regard to PDI, zero value express a perfectly symmetric population, on the other hand, a value of 1 illustrates a completely polydisperse population. All factors revealed no significant impact with *p* values of .11, .24 and .57 for (X_1_), (X_2_) and (X_3_), respectively. The PDI of the measured TPs ranged from 0.35 ± 0.03 to 0.72 ± 0.04 (Table 3). This high PDI is commonly recognized in vesicles assembled by thin-film hydration approach (Salama & Aburahma 2016).

### ZP evaluation

Considering optimal mucus penetration, a nearly neutral nanocarrier surface charge is desirable to maintain mucus penetrating aspects (Lai et al. 2009). However, Karau et al. (1996) suggested that slightly negatively charged vesicles are beneficial for a stable association of positively charged drug to vesicle membrane and prevent drug leakage from the vesicle. ZP of all TPs were negative and ranged from −5.03 ± 0.26 to −12.43 ± 0.30 mV (Table 3). The small ZP values of the fabricated TPs might be correlated to the presence of neutral phospholipid (Engesland et al. 2015). Furthermore, the weakly basic nature of the drug itself as FTN was protonated in the aqueous medium so it might decrease ZP. On the other hand, the addition of SDC imparted a negative charge to the prepared TPs due to its nature as anionic surfactants (Albash et al. 2019). Further, the existence of ethanol might contribute to the negative charge of TPs as ethanol is recognized to form a negative charge (Nasr & Wahdan 2019). It is worth mentioning that there was a slight variation in ZP values, but the change was not significant.

### Selection of the optimal TP

The overall desirability of the optimal TP was 0.67 which proposed to be prepared employing 1:3 as terpenes ratio (X_1_), SDC amount (X_2_) at 10 mg and ethanol concentration (X_3_) of 11.26%. Hence, it was prepared and evaluated. As shown in Table 4, the residuals among the expected and observed responses were small illustrating the adequacy of the optimization step. Thus, the optimal TP was considered promising to be used in further investigation.

**Table 4.** Predicted and observed values of the optimal TP-loaded FTN.

Factor (independent variables)	Optimized level		
X_1_: Terpenes ratio	1:3		
X_2_: SDC amount (mg)	10		
X_3_: Ethanol concentration (v/v)	11.26		
Responses (dependent variables)	Expected	Observed	Residual^a^
Y_1_: EE (%)	62.12	62.18	0.06
Y_2_: PS (nm)	329.22	310.00	−19.22
Y_3_: PDI	0.40	0.20	−0.20

^a^Residual: expected-observed.

EE%: entrapment efficiency percent; FTN: fenticonazole nitrate; PS: particle size; PDI: polydispersity index; SDC: sodium deoxy cholate; TPs: terpesomes.

### Transmission electron microscopy (TEM)

**Figure 3.**
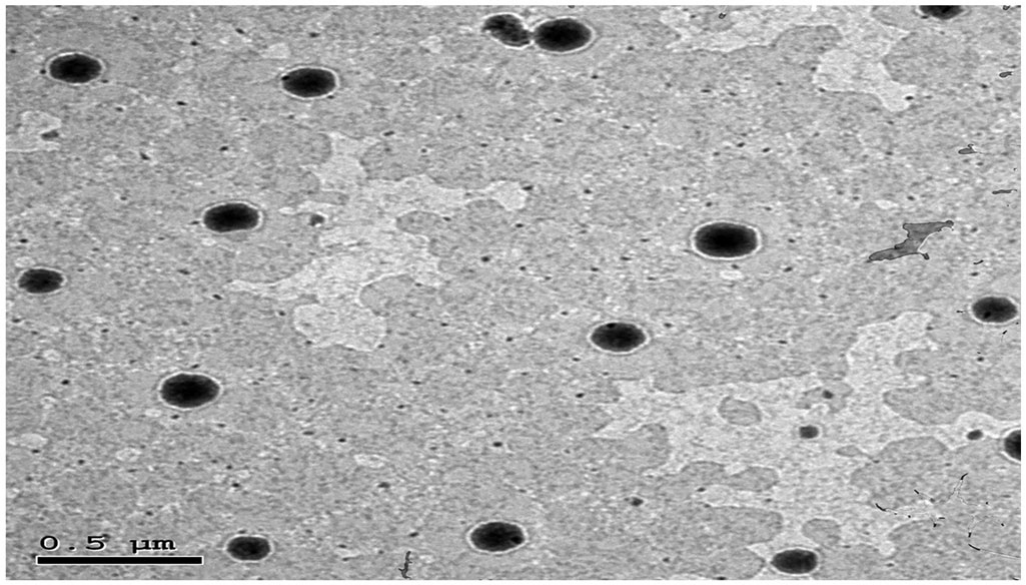
Morphology of the optimal TP. TP: terpesomes.

The structural assessment illustrated that the optimal TP was spherical in shape with uniform size distribution (Figure 3). The PS of the TPs assessed by Zetasizer was in a good agreement with TEM observations.

### Differential scanning calorimetry (DSC)

**Figure 4.**
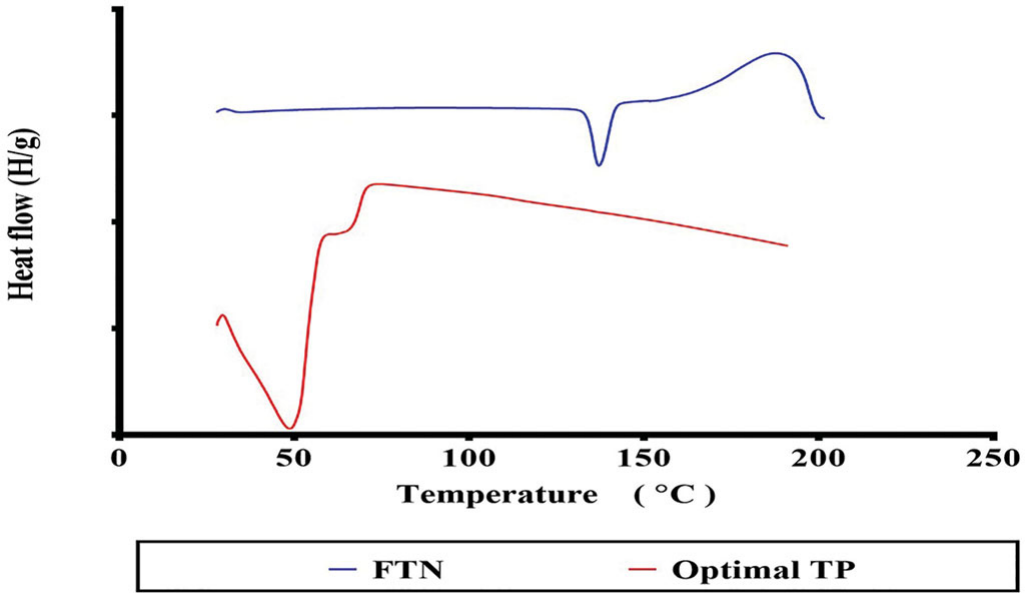
DSC thermograms of FTN and the optimal TP. FTN: fenticonazole nitrate and TP: terpesomes.

From Figure 4, FTN displayed an endothermic peak related to its melting point at 135 °C (Kim et al. 1989). The thermogram of the optimal TP did not show the melting peak for FTN. This indicated that FTN was present in amorphous state and drug was successfully entrapped within the TPs (Albash et al. 2019).

### Elasticity evaluation

The design of mucus invading nanovesicles demands consideration of PS and surface features of the vesicles along with mucus components and its physiological features. In addition, obtaining mucus penetrating nanovesicles as vaginal drug delivery is crucial for extracellular drug liberation where extended vesicle retention in the vaginal tract translates to continuing drug supply. These nanovesicles should invade across both luminal and adherent mucus layers and enter the underlying epithelium before generating their desired therapeutic activity (Wong et al. 2014). The elasticity measurements for classical bilosomes and TPs were 59% and 40%, respectively. The significant (*p* < .05) potency of TPs compared to classical bilosomes might be accounted for the existence of terpenes and ethanol in TPs constructs that might enhance vaginal mucus penetration over classical bilosomes. These results are in agree with Subongkot et al. (2012) who concluded that ultra-deformable liposomes containing terpenes demonstrate greater ability to squeeze themselves through intercellular pathways than plain ultra-deformable liposomes as a result of the synergist effect of the two membrane softeners.

### *Ex vivo* permeation studies

**Figure 5.**
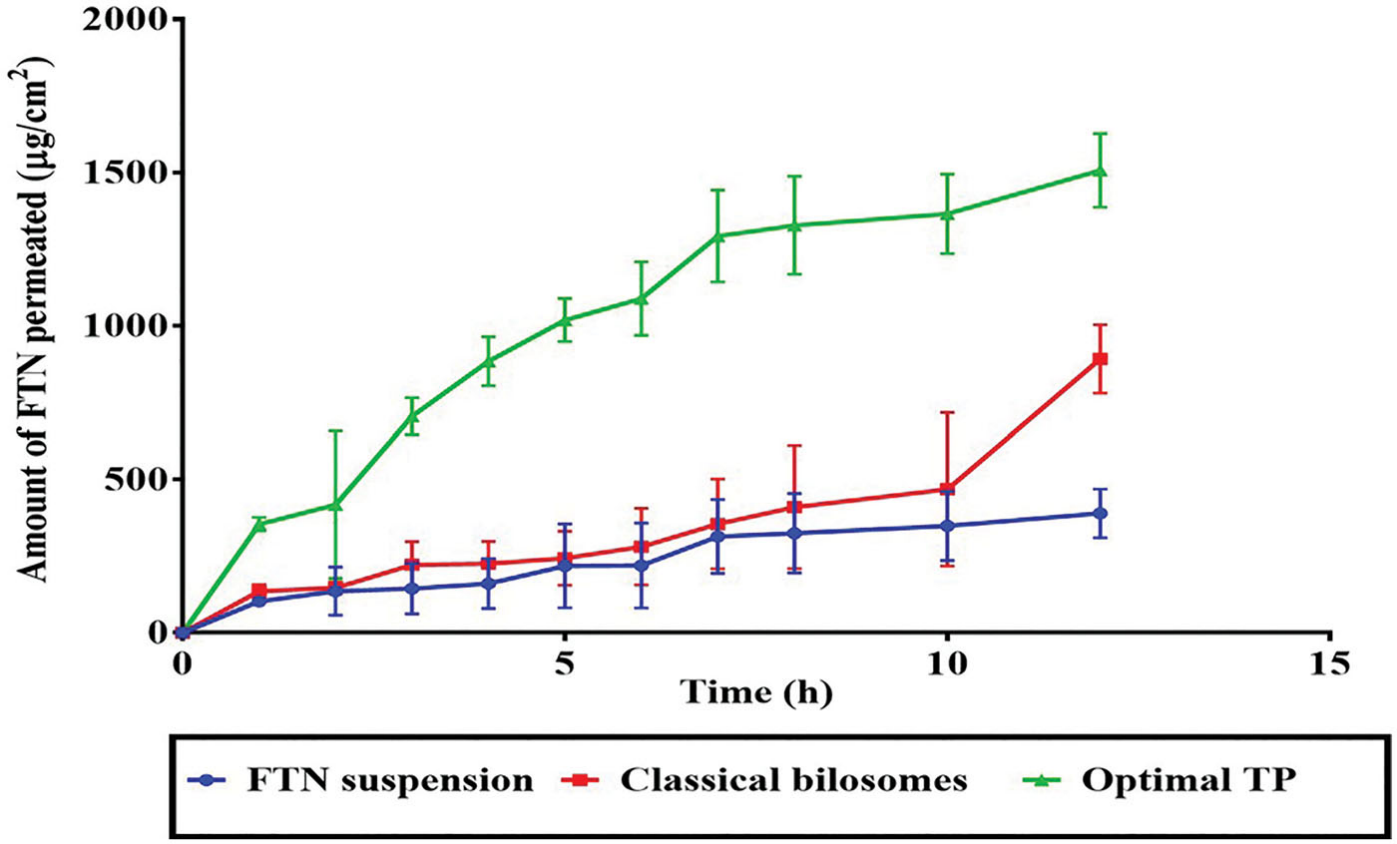
Cumulative amount of FTN permeated per unit area from classical bilosomes and the optimal TP relative to FTN suspension. FTN: fenticonazole nitrate; TP: terpesomes.

Terpenes are considered as a potent infiltration promoter. Further, the existence of ethanol and SDC boosts the deformity of TPs. These phenomena, i.e. increased deformability of TPs is thought to facilitate vesicles mucus penetration (Qadri et al. 2017). As well, vesicles might greatly penetrate mucus inside the vagina, augmenting vaginal drug allocation and retention rather than being cleared rapidly in the outer, luminal layers of the mucus (Ensign et al. 2012). From Figure 5, it is obvious that the amount of FTN infiltered from optimal TP was significantly (*p* < .05) the highest compared to both classical bilosomes and FTN suspension. The ER was 3.65-fold for optimal TP and 2.28 for classical bilosomes in correlation with FTN suspension as depicted in Table 5. From the previous findings, the presence of terpenes and ethanol might enhance the mucus penetrating ability of TPs over classical bilosomes. Same results were obtained from Li et al. (2016) who found the permeation of Fibrauretine was improved by encapsulating the drug in propylene glycol embed ultra derformable liposomes. These results suggested that ultra-deformable liposomes containing permeation enhancer are more effective at enhancing permeation through the vaginal mucosa. It is worthy to mention that the amount of FTN deposited was 76.50 µg/cm^2^, 171.32 µg/cm^2^, and 376.50 µg/cm^2^ from FTN suspension, classical bilosomes, and the optimal TP, respectively. The previous results confirmed that the higher quantity of FTN accumulated and remained in vaginal tissues would form drug reservoir, this could prolong the residence time at the site of administration and in favor of prolonging the drug’s localized activity from the optimal TP compared to both classical bilosomes and FTN suspension.

**Table 5.** Vaginal permeability parameters from FTN suspension, classical bilosomes and the optimal TP.

Vaginal permeability parameters	FTN suspension	Classical bilosomes	Optimal TP
Total amount of FTN permeated per unit area after 12 h (μg/cm^2^)	389.89 ± 79.49	891.68 ± 111.44	1507.09 ± 120.00
J_max_ (μg/cm^2^/h)	46.02 ± 9.38	105.25 ± 13.15	167.89 ± 10
ER	1	2.28 ± 0.78	3.64 ± 0.54

Note: Data are presented as mean ± SD (*n* = 3).

J_max_: Permeation flux; ER: enhancement ratio; FTN: fenticonazole nitrate; TP: terpesomes.

### *In vivo* studies

#### Antifungal study

**Figure 6.**
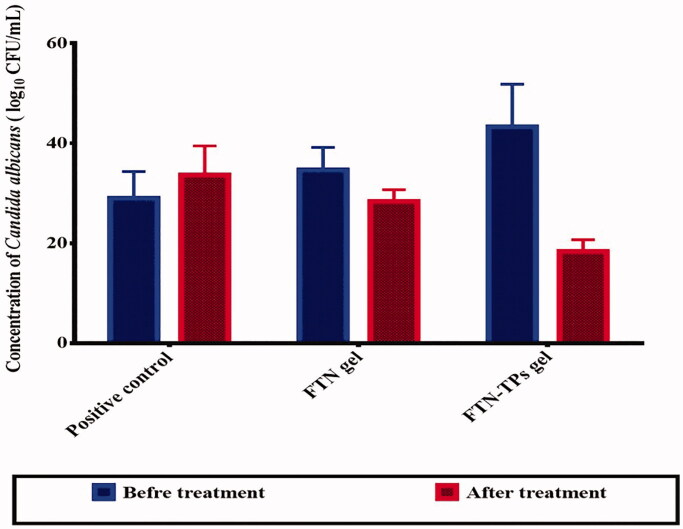
*In-vivo* study graphical chart of positive control, FTN gel and FTN-TPs gel treated groups. FTN: fenticonazole nitrate and TP: terpesomes.

Microscopical examination of Gram stained vaginal washes and the fungal count on Sabouraud dextrose agar plates were used to evaluate the severity of vaginal infection of rats used for the i*n vivo* studies, and to compare the treatment effectiveness of the formulations (Figure 6). Statistical analysis showed that the number of CFU was significantly (*p* < .05) lower for animals treated with FTN-TPs gel in comparison with FTN-gel treated group. It is worth noting that, there was a significant (*p* < .05) difference between FTN-TPs gel and positive control group while in contrast there was no significant (*p*>.05) difference between FTN-gel and positive control group. The previous findings demonstrated that FTN-TPs gel decreased *C.albicans* infection when administered by the intravaginal route compared to FTN-gel. This shows that the FTN-TPs gel was able to promote drug absorption and penetration through the vaginal mucosa due to its high deformability.

#### *In vivo* histopathological study

**Figure 7.**
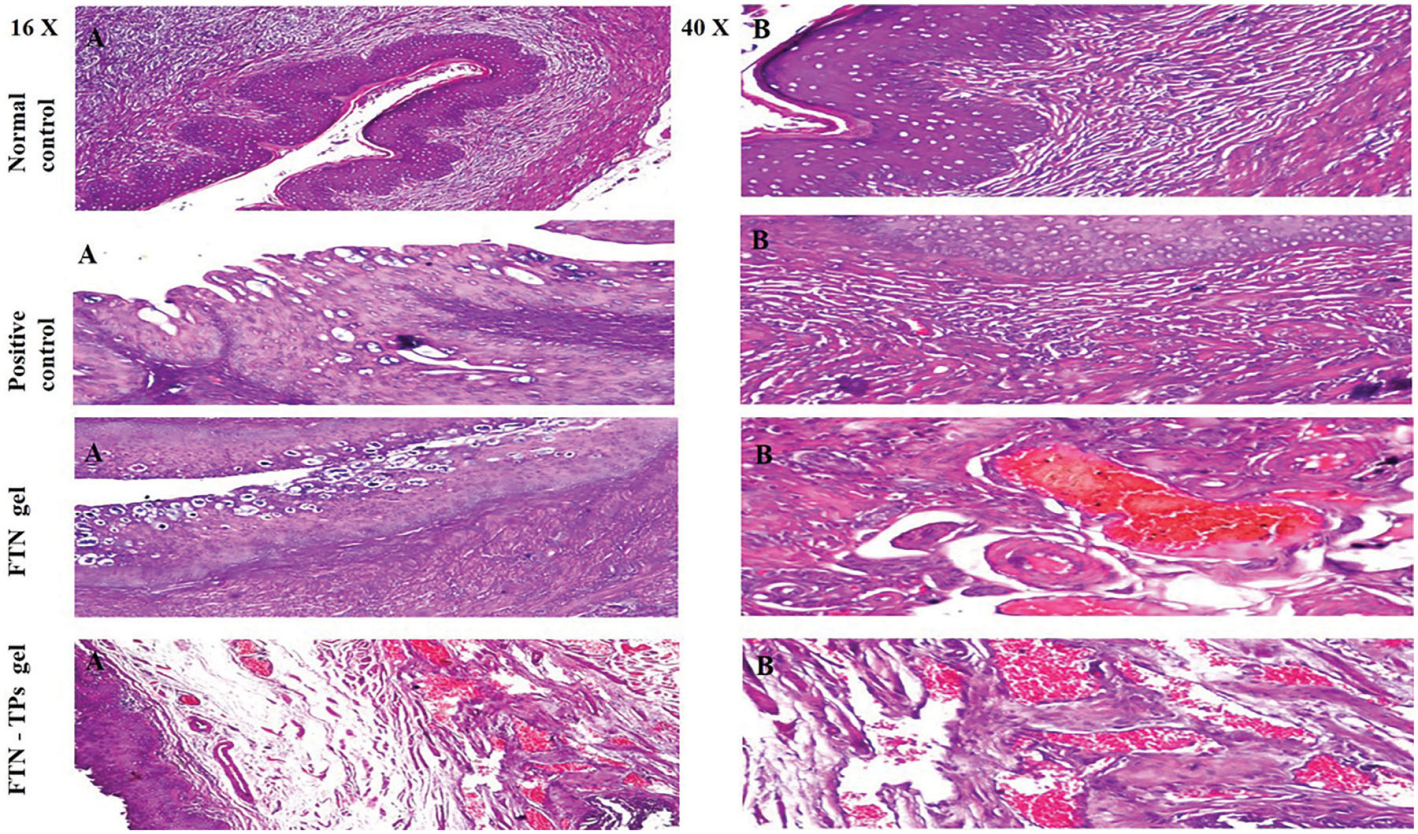
Photomicrographs showing histopathological sections (hematoxylin and eosin stained) of normal control (group I), positive control (group II), rat’s treated with FTN gel (group III) and rat’s treated with FTN-loaded UTPs gel (A) magnification power of 16x and (B) magnification power of 40x. FTN: fenticonazole nitrate; TP: terpesomes.

Figure 7 showed the following, group I showed normal structure of the stratified lining mucosal epithelium and the lamina propria, muscular layer and serosa. For group II, vacuolar degeneration and hyperplasia were detected in the lining stratified mucosal epithelium associated with massive inflammation in the lamina propria of the mucosa as well as in the underlying muscular layer and congestion of the blood vessels in the later. For group III the stratified lining mucosal epithelium showed vacuolar degeneration, while the underlying lamina propria and muscularis were infiltrated by inflammatory cells. In addition, group IV showed edema in the lamina propria of the mucosa with congestion in the blood vessels of the muscular layer besides, the absence of the inflammatory cells indicated the safety and tolerability of FTN-TPs gel for vaginal drug delivery.

## Conclusion

The utilization of TPs with mucus penetrating potency might enhance vaginal delivery of FTN from the perspectives of enhanced drug infiltration into vaginal epithelial mucosa. BBD aided in fabricating the optimal TP that displayed spherical shape, reasonable drug EE% and small PS. Further, *ex vivo* assessment presented the greatest infiltration from the optimal TP over classical bilosomes and FTN suspension. Moreover, the optimal TP was subjected to DSC studies that asserted the entrapment of FTN among the TPs layers. *In vivo* studies showed significant prevention effect in immunocompromised rat model from FTN-TPs gel compared to FTN gel. In addition, histopathological study confirmed the safety of the optimal TP. Taken together, these findings emphasize the high practical value of FTN-TPs gel in the management of vaginal candidiasis.
